# Reducing pediatric total-body PET/CT imaging scan time with multimodal artificial intelligence technology

**DOI:** 10.1186/s40658-023-00605-z

**Published:** 2024-01-02

**Authors:** Qiyang Zhang, Yingying Hu, Chao Zhou, Yumo Zhao, Na Zhang, Yun Zhou, Yongfeng Yang, Hairong Zheng, Wei Fan, Dong Liang, Zhanli Hu

**Affiliations:** 1 Lauterbur Research Center for Biomedical Imaging, Shenzhen Institute of Advanced Technology, Chinese Academy of Sciences, Shenzhen, 518055 China; 2 Department of Nuclear Medicine, Sun Yat-sen University Cancer Center, Guangzhou, 510060 China; 3 United Imaging Healthcare Group, Central Research Institute, Shanghai, 201807 China

**Keywords:** PET/CT, Multimodal artificial intelligence techniques, Pediatric, Short scan time

## Abstract

**Objectives:**

This study aims to decrease the scan time and enhance image quality in pediatric total-body PET imaging by utilizing multimodal artificial intelligence techniques.

**Methods:**

A total of 270 pediatric patients who underwent total-body PET/CT scans with a uEXPLORER at the Sun Yat-sen University Cancer Center were retrospectively enrolled. ^18^F-fluorodeoxyglucose (^18^F-FDG) was administered at a dose of 3.7 MBq/kg with an acquisition time of 600 s. Short-term scan PET images (acquired within 6, 15, 30, 60 and 150 s) were obtained by truncating the list-mode data. A three-dimensional (3D) neural network was developed with a residual network as the basic structure, fusing low-dose CT images as prior information, which were fed to the network at different scales. The short-term PET images and low-dose CT images were processed by the multimodal 3D network to generate full-length, high-dose PET images. The nonlocal means method and the same 3D network without the fused CT information were used as reference methods. The performance of the network model was evaluated by quantitative and qualitative analyses.

**Results:**

Multimodal artificial intelligence techniques can significantly improve PET image quality. When fused with prior CT information, the anatomical information of the images was enhanced, and 60 s of scan data produced images of quality comparable to that of the full-time data.

**Conclusion:**

Multimodal artificial intelligence techniques can effectively improve the quality of pediatric total-body PET/CT images acquired using ultrashort scan times. This has the potential to decrease the use of sedation, enhance guardian confidence, and reduce the probability of motion artifacts.

**Supplementary Information:**

The online version contains supplementary material available at 10.1186/s40658-023-00605-z.

## Introduction

PET/CT is an indispensable technology for diagnosing malignant tumors [1–4], which are generally not localized but often systemic [5–7]. Therefore, PET/CT is usually used to obtain a whole body scan to discover not only the primary site of lesion but also any metastatic lesions in soft tissue organs and bones throughout the body. PET uses radioactive tracers, special cameras and computers to image tracer distribution and evaluate organ and tissue functions. Typically, tracer administration activity and data acquisition time are positively correlated with imaging quality [8, 9]. Longer acquisition times may introduce motion artifacts in the images, especially in young children who are still developing and have more frequent limb movements. Such motion artifacts will lead to missed diagnoses or misdiagnoses for smaller lesions [10].

Sedation is often administered to young children to ensure that they remain relatively motionless throughout the examination. However, sedation can lead to numerous potential short-term side effects, and failed sedation contributes significantly to guardian dissatisfaction with the child's sedation experience [11]. Imaging using short-term scan data can reduce the dose of sedatives and decrease potential artifacts that can confound the image diagnosis [12]. However, there is a trade-off between image quality and radiation exposure, and the use of short-term scans usually means that high doses of radiopharmaceuticals need to be injected. To address this trade-off, PET scanner hardware and software continue to undergo improvements [13–15]. Recently, a new PET scanner called uEXPLORER was introduced. It has an axial FOV of 194 cm, allowing for total-body imaging with just one bed position, and its effective sensitivity is enhanced approximately 40-fold [3, 14, 16–20].

The data acquired from short-term scans at standard tracer doses carry a large amount of noise, which is very difficult to suppress using conventional reconstruction algorithms. Recently, deep learning has shown excellent performance in low-dose PET imaging. Convolutional neural network (CNN) and generative adversarial network (GAN) models have been successfully used to reconstruct near full-dose PET images from low-dose data [21–25]. CNNs based on fused multimodal data have been shown to combine the advantages of the data of each modality, which can effectively and significantly reduce the tracer dosage [26–30].

In this retrospective study, we investigated whether artificial intelligence algorithms can contribute to reducing the scan time by predicting full-time images from short-term scan images. We also investigated whether deep learning framework models that fuse multimodal image data (CT prior information) perform better than networks based on single-modal data.

## Materials and methods

The data of this retrospective study came from the Sun Yat-sen University Cancer Center. The study was approved by the institutional review board of the center, and informed consent was obtained from all of the patients' legal guardians.

### Data acquisition

**Fig. 1:**
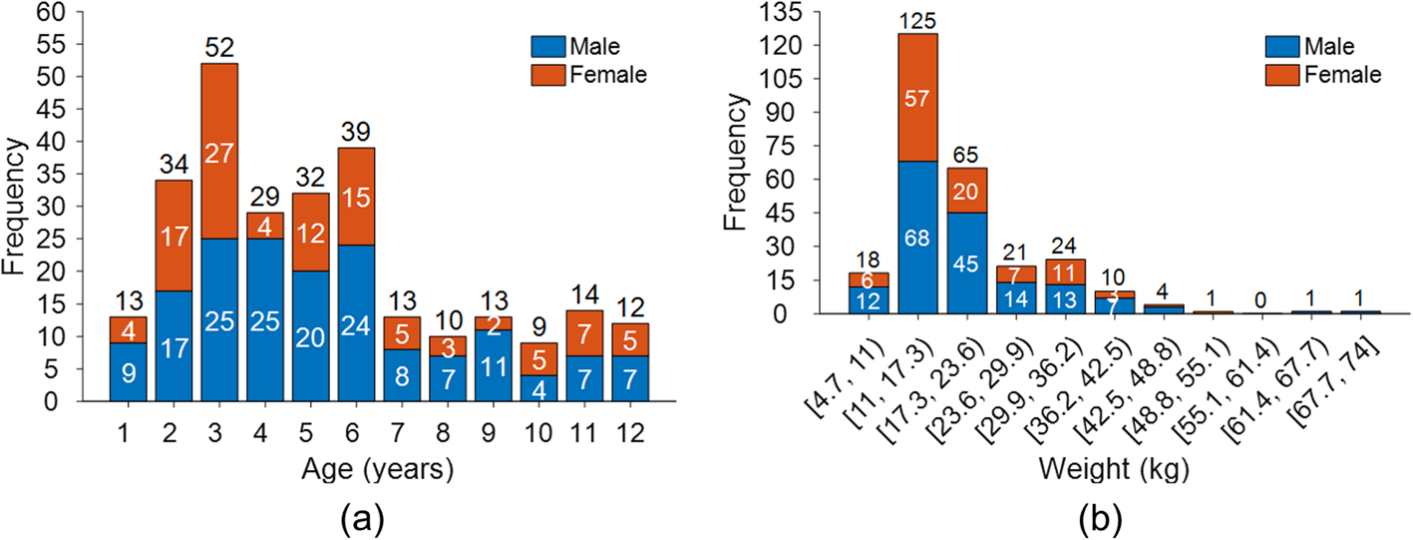
Distribution of age, weight and sex of the 270 children enrolled in the study. **a** Age and sex distribution. **b** Weight and sex distribution

A total of 270 pediatric patients who underwent total-body PET/CT using the uEXPLORER scanner (uEXPLORER, United Imaging Healthcare) at the Sun Yat-sen University Cancer Center from July 2020 to April 2022 were retrospectively enrolled in this study (Fig. 1) (median age 5 years, range [1, 12]; median weight 17 kg, range [4.7, 74]). The clinical characteristics of the patients are summarized in Table 1. The inclusion criteria were as follows: age < 13 and body weight < 75 kg, and the exclusion criteria were waiting time after ^18^F-FDG injection > 75 min and no suspected FDG-avid lesions.

**Table 1:** Patient clinical characteristics

Pathological type	Number of patients
Non-Hodgkin’s lymphoma	58
Hodgkin’s lymphoma	10
Rhabdomyosarcoma	57
Nephroblastoma	45
Nasopharyngeal carcinoma	24
Ewing's sarcoma	16
Germ cell tumor	13
T-cell acute lymphoblastic leukemia	11
Myxoid liposarcoma	11
Schwannoma (neurilemoma)	9
Langerhans cell histiocytosis	8
Alveolar soft-part sarcoma	5
Osteosarcoma	3

One hundred and twenty patients were selected as the evaluation dataset (Additional file 1: Fig. S2); the data of these patients were not included in the training of the network. For the remaining data from 150 patients (Additional file 1: Fig. S1), we adopted a K-fold cross-validation strategy (K = 10) to account for the lack of training samples. The dose of ^18^F-FDG was approximately 3.7 MBq/kg (3.7 ± 0.37 MBq/kg) (Additional file 1: Table S1), and the acquisition time was 600 s. Low-dose total-body CT scans were acquired with a dynamically adjusted tube current and 100 kV tube voltage (rotation time 0.5 s, pitch 1.0125, collimation 80 × 0.5 mm) and were reconstructed in a 512 × 512 matrix for PET attenuation correction. PET images were reconstructed using TOF-OSEM with the following parameters: PSF modeling, 3 iterations, 20 subsets, matrix 256 × 256, slice thickness 2.89 mm, voxel size 2.34 × 2.34 × 2.89 mm^3^, Gaussian postfiltering (3 mm), and all necessary correction methods, including scattering and attenuation corrections.

### Image preprocessing

List mode PET data with an acquisition time of 600 s were reconstructed as full-time ground-truth images. The PET images of the short-term scans were simulated by truncating the list-mode data. The first 6 s, 15 s, 30 s, 60 s and 150 s of the list-mode data were truncated for reconstruction using the same protocol reported in the previous subsection. For simplicity, the image series reconstructed with 6- to 600-s data are referred to as the G6s, G15s, G30s, G60s, G150s and G600s groups in this paper. The CT images were registered to the PET images using MATLAB (MathWorks, Natick, MA) software. All images were resampled to the voxel dimensions of the acquired PET volumes. The intensities of all reconstructed PET images were normalized to the 0–1 range using the maximum standardized uptake value across all patient data. The intensities of all low-dose CT images were also normalized to the 0–1 range using the maximum HU value in all patient data.

### CNN implementation

**Fig. 2:**
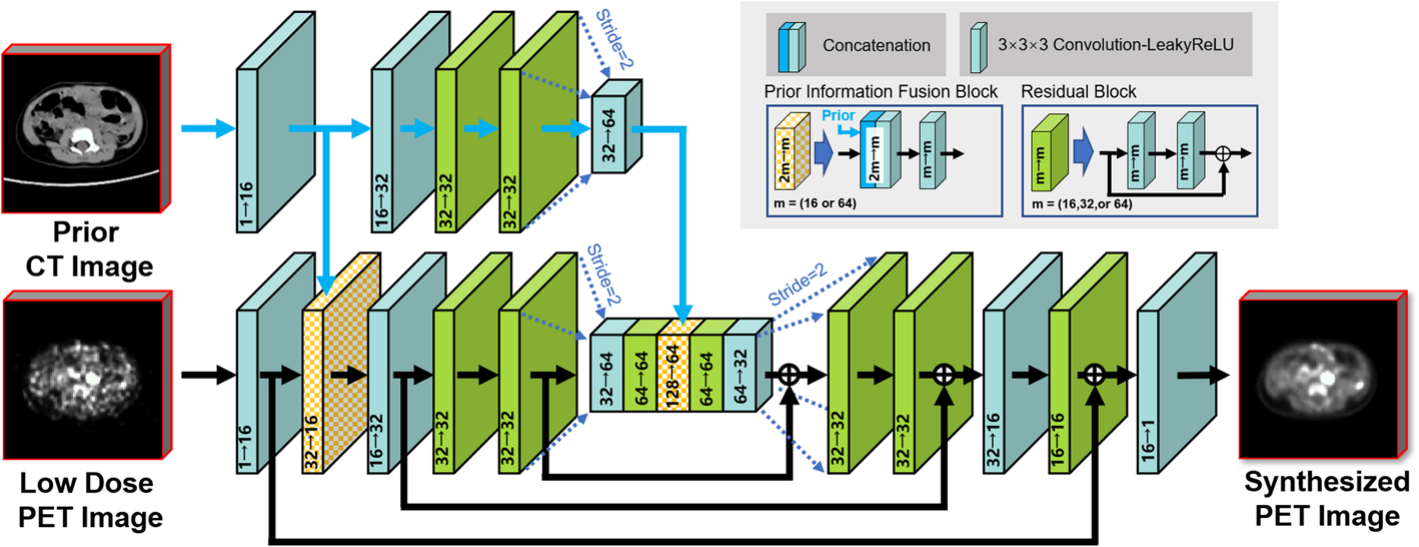
Schematic diagram of the 3D convolutional neural network (p3DNet) used in this work. Two modal-specific encoders and one decoder that synthesizes the full-dose PET images are included. The arrows indicate the flow of computational operations, and the number of input and output feature images for each module is marked below its box

The proposed 3D neural network is shown in Fig. 2. The main feature of the network is the use of multimodal data as input to generate single-modal data. Based on an investigative assessment of different state-of-the-art deep learning structures, including ResNet [31] and U-Net [32], we adopted the 3D U-Net encoder–decoder architecture strategy with the residual module as the main framework for the network. Fusion from the high-dimensional features of the individual modal images can lead to better integration of complementary information in each modality [33, 34]. Therefore, we used the high-dimensional features extracted from the CT images after multiple 3D convolutional layers as the prior information introduced into the encoder of the network.

The input and output of the 3D network were multislice data of size *H* × *W* × *S*, where *H* and *W* denote the image height (256) and width (256), respectively, and S denotes the depth of successive adjacent multislice data. To reduce the computation time and memory consumption, we fix S to 5. The encoder and decoder part consisted of 3D convolutional layers (using 3 × 3 × 3 filters) and a leaky rectified linear unit (LeakyReLU) activation function. The number of channels is labeled below each box in Fig. 2. The downsampling operation was implemented by a convolutional layer with stride = 2. Correspondingly, the upsampling operation was implemented by a deconvolutional layer that scaled the image size by a factor of two. Skip connections were applied between the residual module in the network and the encoder–decoder layer at symmetric positions to preserve the feature information. In the encoder–decoder component, the combined feature maps from the encoder component and the upsampling component were passed to the corresponding decoder component, thus increasing the information diversity.

The network was trained with the images from all short-term scans. To enhance the network's ability to recover anatomical structures and texture details, the loss function of the network was a combination of L2 normal and perceptual loss [35]. The network was trained with a batch size of 5 over 400 epochs, and the initial learning rate was 3 × 10^−4^. The network was constructed using the PyTorch deep learning framework on an Ubuntu 16.04 system with a Titan 2080Ti GPU and was optimized using the Adam optimizer with a cosine annealing strategy to speed up convergence [36, 37].

### Reference methods

The proposed 3D network with fused CT prior information was named p3DNet. The nonlocal means (NLM) method [38] and the same 3D network without the fused CT prior information, named 3DNet, were used as reference methods. The search window and patch sizes of the NLM method were 27 × 27 and 3 × 3, respectively. The main frameworks of 3DNet and p3DNet are the same except for the prior information fusion block, which is described in detail in Additional file 1: T1.

### Quantitative imaging analysis

The image quality was evaluated by an experienced technician under the supervision of a radiologist. The images generated by the neural network were first visually inspected for artifacts. Afterward, the images were restored to their original values according to the normalized parameters of the previous preprocessing process. The performance of the methods was evaluated using two computational metrics in computer vision, including the peak signal-to-noise ratio (PSNR) and structural similarity index (SSIM).

These metrics are defined as follows:

1 $$\begin{array}{*{20}c} {{\text{PSNR}} = \frac{10}{{\log 10}}\log \frac{{V^{2} }}{{\frac{1}{MN}\mathop \sum \nolimits_{i,j}^{MN} \left( {x_{r} \left( {i,j} \right) - x\left( {i,j} \right)} \right)^{2} }}} \\ \end{array}$$

where ${{\varvec{x}}}_{{\varvec{r}}}$ is a full-time, high-dose image of size *M* × *N*, and ${\varvec{x}}$ is the image to be measured. *V* denotes a scalar and denotes the maximum value of the evaluated image ${\varvec{x}}$.

2 $$\begin{array}{*{20}c} {{\text{SSIM}} = \frac{{\left( {2\mu_{x} \mu_{{x_{r} }} + a_{1} } \right)\left( {2\sigma_{{x,x_{r} }} + a_{2} } \right)}}{{\left( {\mu_{x}^{2} + \mu_{{x_{r} }}^{2} + a_{1} } \right)\left( {\sigma_{x}^{2} + \sigma_{{x_{r} }}^{2} + a_{2} } \right)}}} \\ \end{array}$$

where ${\mu }_{x}$ and ${\sigma }_{x}^{2}$ denote the mean value and variance of the evaluated image ${\varvec{x}}$, respectively, and similar properties were defined for the reference image ${x}_{r}$. ${\sigma }_{x,{x}_{r}}$ are the covariance values of x and ${x}_{r}$. ${a}_{1}$ and ${a}_{2}$ are the two constants used to stabilize divisions with weak denominators, and they are usually fixed at 1 × 10^–6^ and 3 × 10^–6^.

Five two-dimensional circular regions of interest (ROIs) with a diameter of 2 cm were drawn over homogeneous regions of the liver parenchyma of the 120 patients in the evaluation set, with care to avoid blood vessels and intrahepatic lesions to record semiquantitative uptake measurements of the liver, including SUVmax, SUVmean and standard deviation (SD). The smallest measurable suspicious lesion (not necessarily malignant) with the shortest long diameter was identified, and the ROI of this lesion was drawn on the slice with the maximum lesion diameter to measure SUVmax. The SUVmax of the lesion (on PET image) was documented. The lesion-to-background ratio (LBR) was calculated by dividing the SUVmax of the lesion by the SUVmean of the liver. The semiquantitative metrics obtained by different methods for different short scan time groups were compared with those of the G600s images. All ROIs were drawn on the G600s images and transferred to the other groups to ensure that the location and size of the ROI were identical across all groups.

### Qualitative imaging assessment

A subjective assessment of the PET image quality was independently rated by two nuclear radiologists (a senior radiologist with > 10 years of experience and a radiologist with > 5 years of experience) based on a 5-point Likert scale. All patients in the assessment set were read on the volume data, and all datasets (original, postprocessed and neural network synthesized) were anonymized. A 5-point Likert scale was used to evaluate three aspects: (1) the conspicuity of the organ anatomical structures, (2) the conspicuity of the major suspected malignant lesions and (3) the image noise. The status read from the full-scan time images was treated as the ground truth. For each PET image, the physician assigned an image quality score on a five-point scale: 1, uninterpretable; 2, poor; 3, adequate; 4, good; and 5, excellent. The average scores of all readers for each image were calculated, and then the scores of the short-term scan images were compared with the scores of the full-time scan images.

### Statistical analysis

The two-dimensional correlation coefficients between the full-time scan reference images and the images processed with different methods were calculated to determine the concordance between the images. The statistical analysis was performed with the R Statistical package (R4.2.3, the R Foundation) and Microsoft Excel. Paired t tests were used to compare the objective image values (SUVmax, SUVmean and LBR) and the quality metric values (PSNR and SSIM) between two image series created with different algorithms. To overcome the individual change in SUV induced by patient metabolism, the paired t test was corrected with the Bonferroni correction method. The Kruskal‒Wallis rank-sum test and Tukey’s post hoc test for multiple comparisons were applied in subjective image quality analyses between different scan-time groups. A* p* value of < 0.05 was considered to indicate statistical significance.

## Results

### Image quality

**Fig. 3:**
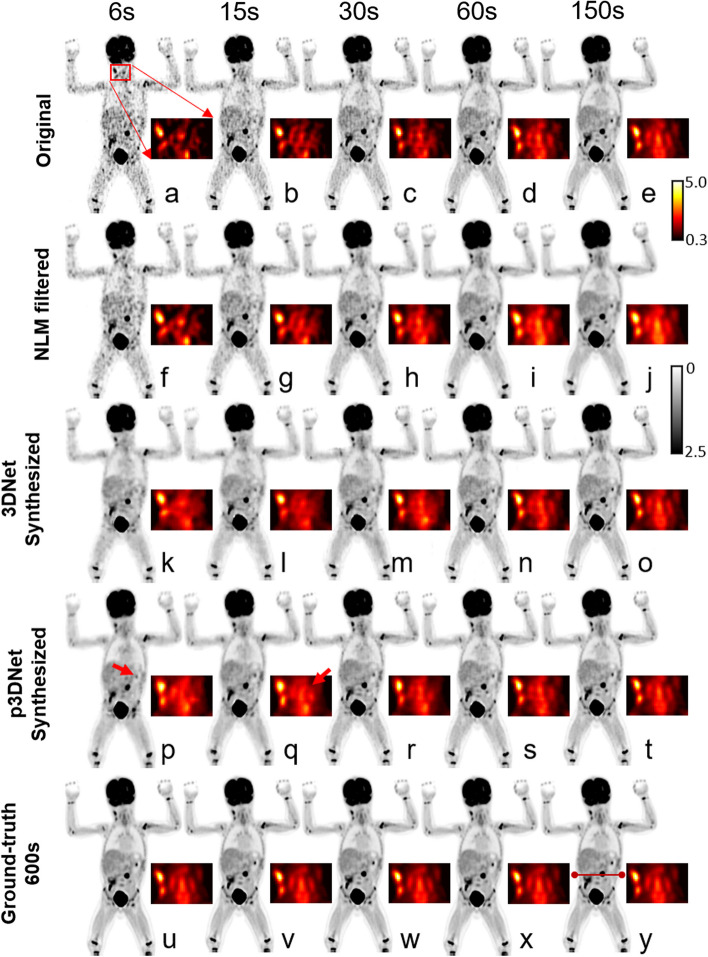
^18^F-FDG PET images processed using different methods taken from a 2-year-old male patient weighing 10 kg with metabolic activity in the left abdominal wall after surgery for a left testicular yolk sac tumor. **a**–**e** PET images at scan times of 6 s, 15 s, 30 s, 60 s and 150 s, respectively, are shown in axial view. **f**–**j** PET images synthesized from a-e using the NLM method. **k**–**o** PET images synthesized from **a**–**e** using 3DNet. **p**–**t** PET images synthesized from a-e using p3DNet. **u**–**y** Full-time reference images. PET images synthesized by the p3DNet method show improved preservation of tumor and tissue structure (red arrows)

**Fig. 4:**
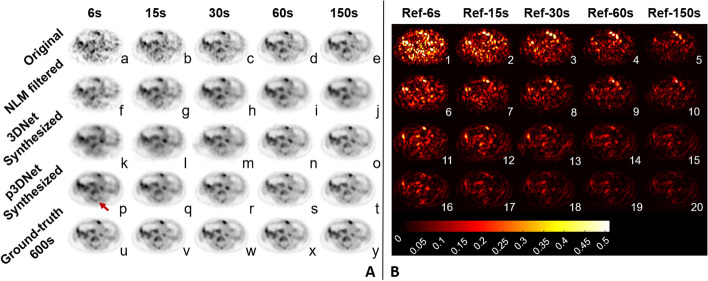
Axial views of the patient’s lesion in Fig. 3 (as indicated by the red line in y in Fig. 3). **a**–**e** PET images at scan times of 6 s, 15 s, 30 s, 60 s and 150 s, respectively, are shown in axial views. **f**–**j** PET images synthesized from **a**–**e** using the NLM method. **k**–**o** PET images synthesized from a-e using 3DNet. **p**–**t** PET images synthesized from a-e using p3DNet. **u**–**y** Full-time reference images. PET images synthesized using the p3DNet method can effectively recover anatomical structures (red arrow). (1–20) are the residual maps (absolute values) obtained by subtracting the reference image from **a**–**t**. (1–5) Residual maps of **a**–**e**. (6–10) Residual maps of (**f**–**j**). (11–15) Residual maps of **k**–**o**. (16–20) Residual maps of (**p**–**t**)

Figure 3 shows ^18^F-FDG PET images processed using different methods taken from a 2-year-old male patient weighing 10 kg with metabolic activity in the left abdominal wall after surgery for a left testicular yolk sac tumor. Based on the image appearance, we can see that the proposed method generates images with preserved tumor and tissue structure (indicated by red arrows) and less noise than other methods. The axial views of the patients’ lesions generated by the different methods, as well as the residual maps between the images and the reference images from the full-time scan, are shown in Fig. 4. The deep learning method p3DNet, which fuses CT prior information, effectively recovers anatomical structures (red arrow) and has the smallest residual values under different short-term scan conditions, indicating good consistency with the reference image.

**Fig. 5:**
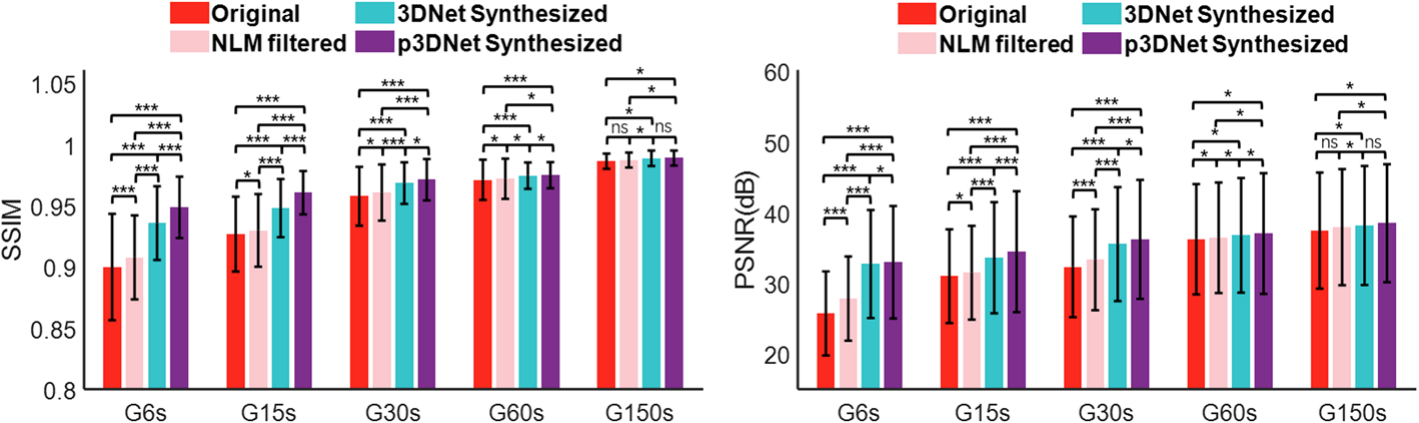
Image quality metrics (SSIM and PSNR) compared among different methods and scan times. The model that synthesizes PET images by fusing prior CT information (p3DNet) is superior in metrics such as SSIM and PSNR. *, ***, and ns indicate *p* < 0.05, *p* < 0.001, and nonsignificant, respectively

The average SSIM and PSNR values calculated from the short-term scan images and the synthesized images relative to the full-time scan images for all the patients in the evaluation set are shown in Fig. 5. The PSNR and SSIM metrics of the images processed by the proposed p3DNet method were significantly greater than those of the original short-term scanned images (*p* < 0.05) and those obtained by using the NLM method (*p* < 0.05) and the 3DNet method (*p* < 0.05) in the G6s, G15s, G30s and G60s groups. When the scan time was extended to 150 s, the difference in the results between the two deep learning-based methods was no longer significant (*p* > 0.05), indicating that the benefit from the CT prior information was not obvious at this scanning duration and that the method proposed in this paper is more advantageous under shorter-term scan conditions.

**Fig. 6:**
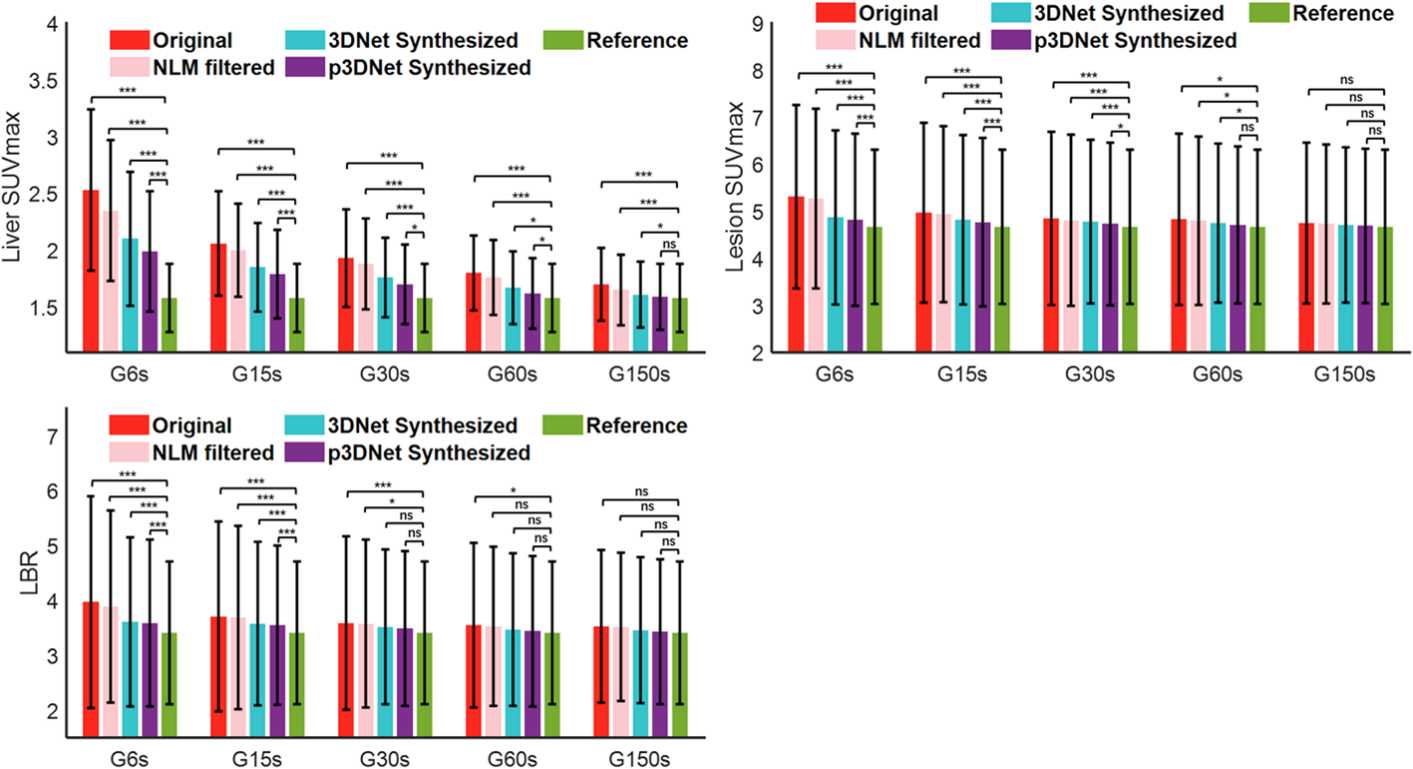
Semiquantitative metrics (Liver SUVmax, Lesion SUVmax and LBR) compared among different methods and scan times. PET images synthesized by the model fusing prior CT information (p3DNet) did not have significant differences in Lesion SUVmax and LBR metrics from the reference image at a scan time of 60 s. *, ***, and ns representing *p* < 0.05, *p* < 0.001, and nonsignificant, respectively

Figure 6 shows the objective measurements of image quality, including SUVmax for liver uptake, SUVmax for lesion uptake, and LBR, using the G600s measurements as a reference. The SUVmax and LBR values of the images synthesized by the p3DNet method are closest to those of the full-time scan reference image for the different short-term scans. The lesion SUVmax and LBR metrics of the PET images synthesized by the model fusing prior CT information (p3DNet) were not significantly different from those of the reference images at a scan time of 60 s (*p* > 0.05). At a scan time of 150 s, the liver SUVmax, lesion SUVmax and LBR metrics were not significantly different from those of the reference image (*p* > 0.05). Semiquantitative metrics (liver SD and lesion SD) are presented in the Additional file 1: Fig. S3. Additional file 1: Fig. S4 shows the Bland‒Altman plots of the change in liver SUVmean from that of G6s, G15s, G30s, G60s, and G150s to G600s. The results showed that the images synthesized by the p3DNet method had the smallest bias and lowest variance among all short-time scan groups relative to the reference standard full-time scan images.

**Fig. 7:**
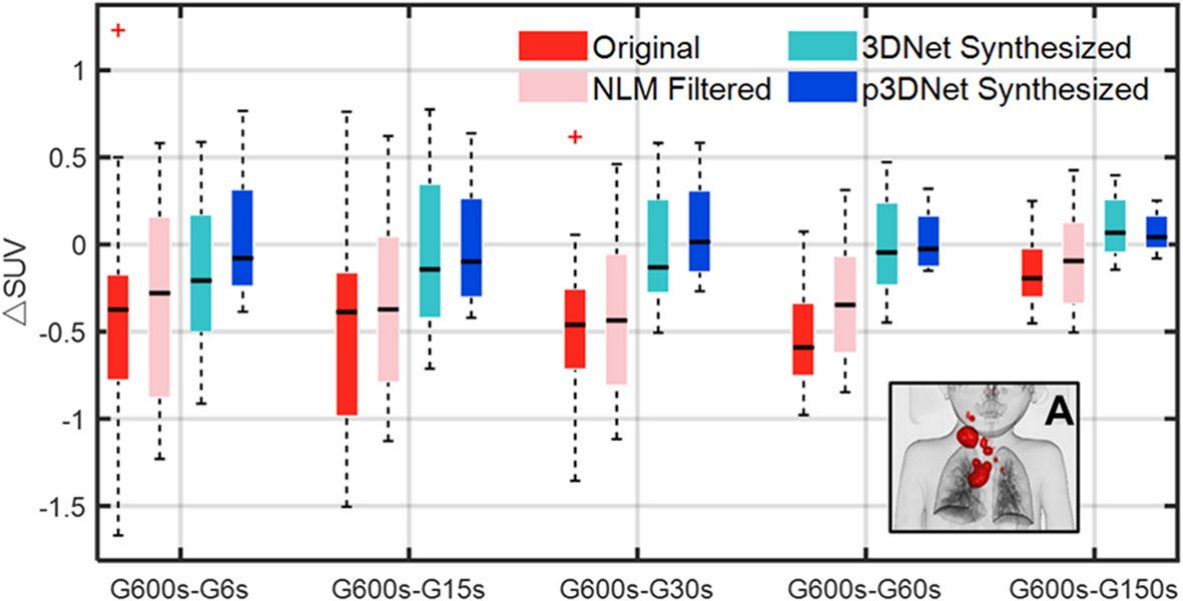
Boxplot of the SUV difference (ΔSUV) distribution of lesion locations in a patient with bilateral cervical mediastinal lymphoma. ΔSUV was calculated by subtracting the images obtained by the different methods from the full-time scan images. The results of the p3DNet method are closest to the full-time scan results for all short-term scan durations

Figure 7 shows a boxplot of the distribution of the SUV difference between the results obtained by the different methods and the full-time scan for a patient with bilateral cervical mediastinal lymphoma lesions. As shown in the red area of panel A in Fig. 7, the analyzed data included all lesion areas in the neck and chest. A mask of the lesion regions was constructed in the volume data of the full-time scan using a segmentation threshold of SUV = 5. Afterward, the mask was applied to the volume data of all conditions, and 2455 voxels were extracted from all lesion regions per volume data. As seen from the figure, the distribution of the data obtained by the deep learning method fusing the prior CT information (p3DNet) is the closest to that of the reference image (full-time scans) for all short-term scans.

Table 2 shows the average two-dimensional correlation coefficients between the images processed by the different methods and the reference full-time scan image. Higher correlation coefficients indicate better concordance between the images. The p3DNet method achieved better results for all short-term scan durations, achieving correlation coefficients of 0.9925 and 0.9971 for G60s and G150s, respectively.

**Table 2:** Average two-dimensional correlation coefficients between the reference full-time scan images and the images processed with different methods

	G150s	G60s	G30s	G15s	G6s
Original	0.9955 ± 0.0092	0.9898 ± 0.0131	0.9818 ± 0.0174	0.9672 ± 0.0213	0.9294 ± 0.0339
NLM	0.9958 ± 0.0085	0.9903 ± 0.0123	0.9822 ± 0.0167	0.9676 ± 0.0201	0.9299 ± 0.0324
3DNet	0.9969 ± 0.0077	0.9918 ± 0.0116	0.9838 ± 0.0161	0.9689 ± 0.0193	0.9310 ± 0.0309
p3DNet	0.9971 ± 0.0077	0.9925 ± 0.0115	0.9844 ± 0.0160	0.9696 ± 0.0191	0.9315 ± 0.0304

### Clinical readings

**Fig. 8:**
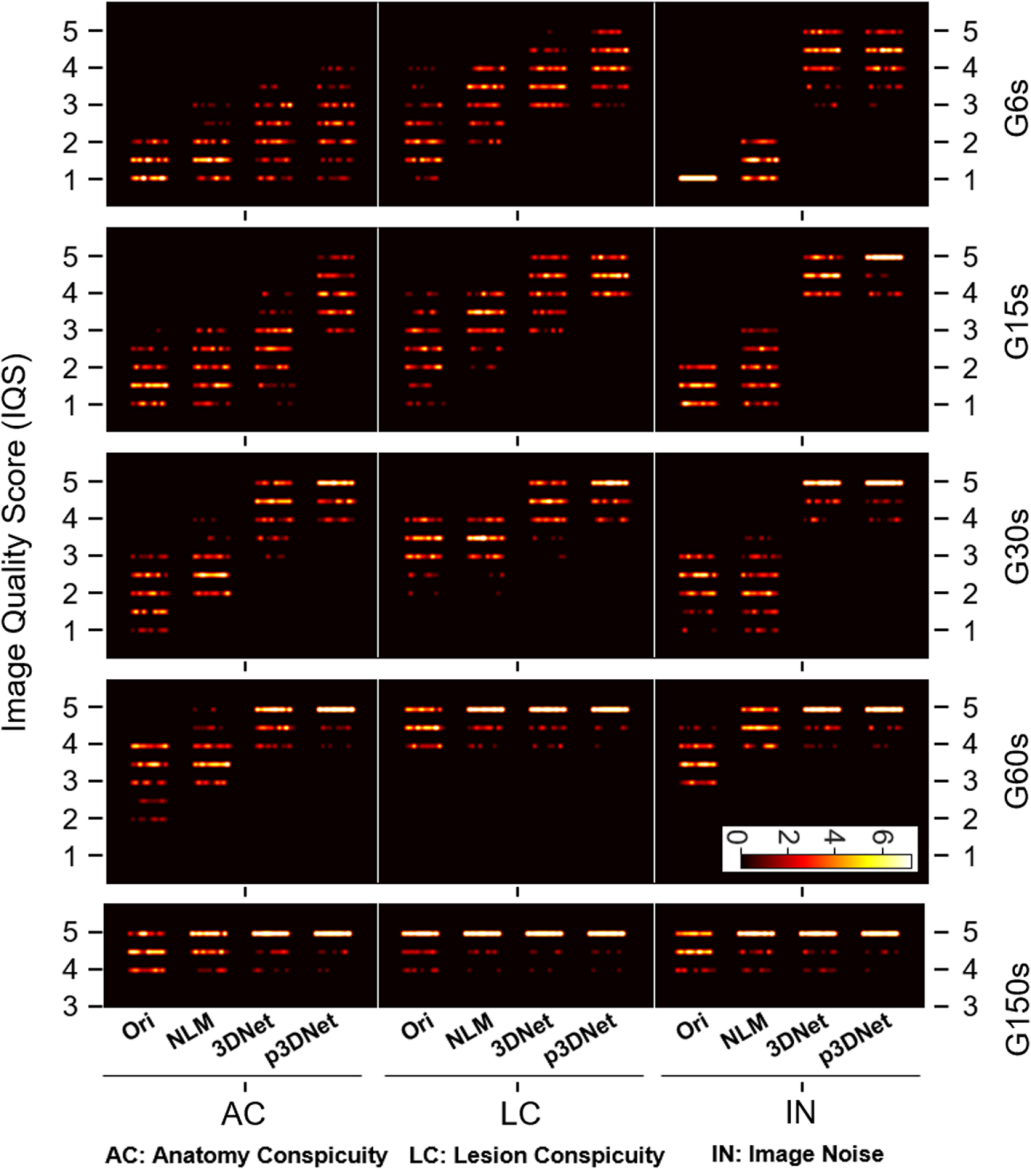
Scatter plots of the average score for different subjective metrics for the images of the 120 patients in the evaluation set, with the anatomical conspicuity (AC) metric in the left column, the lesion conspicuity (LC) metric in the middle column, and the image noise (IN) metric in the right column. The subcolumns from left to right are the average scores of two readers’ subjective ratings of the original images and the images processed by the NLM method, 3DNet and p3DNet, respectively. (There are a total of 120 points for each metric. The brightness of the region is proportional to the concentration of the points. The 600 s group achieved scores of 5 for all metrics)

**Fig. 9:**
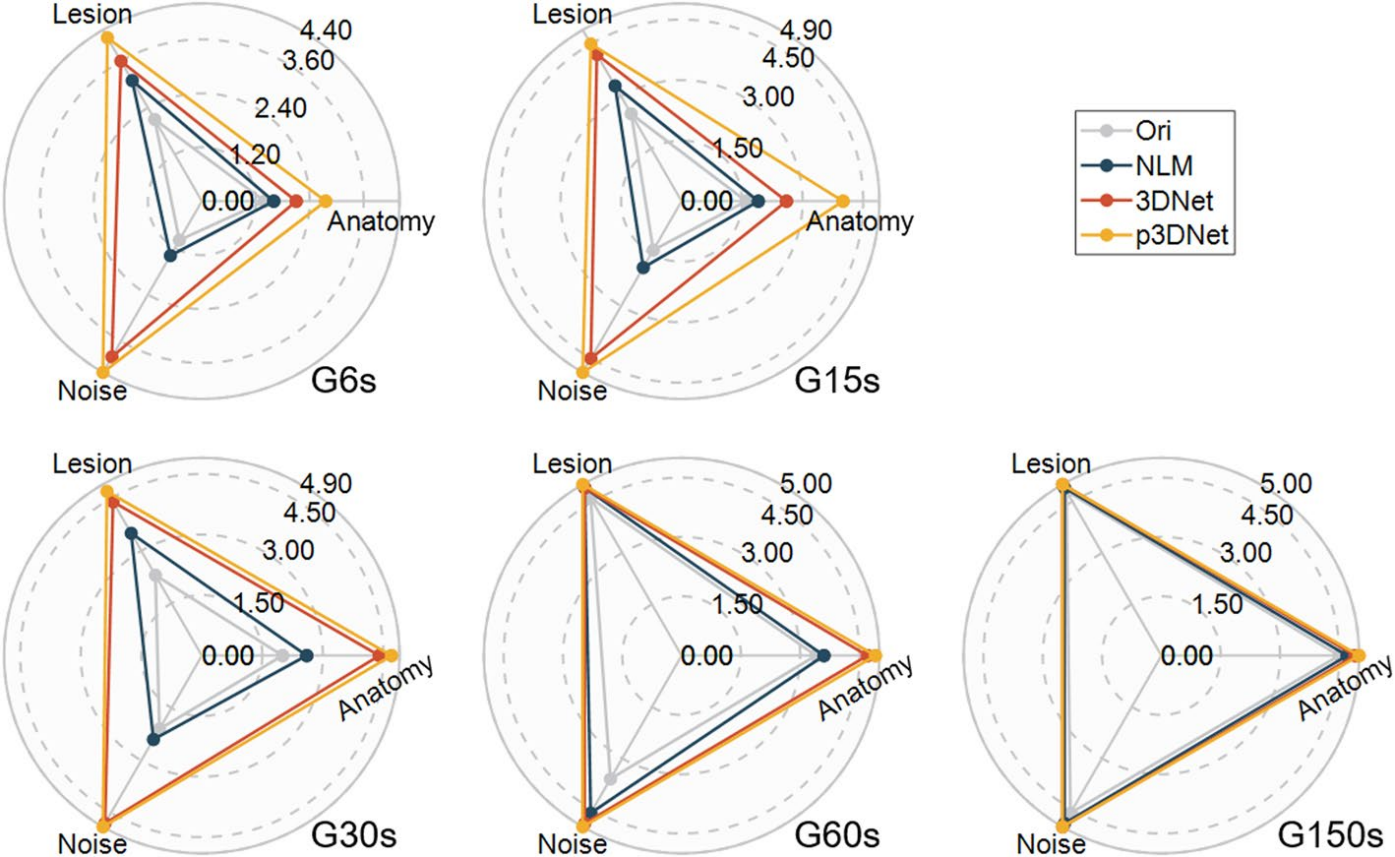
Radar plots of the subjective image quality scores for the different methods in the different dose groups, including the lesion conspicuity scores, anatomy conspicuity scores, and image noise scores

The average subjective image quality scores of the volume data for each patient were calculated by all readers and compared between methods at different dose groups. Figure 8 shows a scatter plot of the average scores of the two readers for different subjective metrics for the images of the 120 patients in the evaluation set, with the anatomy conspicuity (AC) metric in the left column, the lesion conspicuity (LC) metric in the middle column, and the image noise (IN) metric in the right column. The left-to-right subcolumns in each column are the average scores of the subjective ratings of the two readers for the original short-term scanned image and the images processed by the NLM method, 3DNet, and p3DNet. The average scores for the different metrics are shown in Fig. 9 and the Additional file 1: Table S2. From Fig. 8 and Fig. 9, it can be seen that the deep learning-based method has obvious advantages in noise suppression, and the majority of the AC, LC and IN index scores are 5 for the 60-s data acquisition group (G60s). The subjective scores of the images obtained with different methods under different scan times are statistically significant for all metrics; specifically, the images processed by the deep learning methods (3DNet and p3DNet) in the G6s, G15s and G30s groups are significantly different from the images processed by the conventional method (NLM) and the original images (*p* < 0.05), while there is no significant difference in the IN metrics among the deep learning methods (*p* > 0.05). There was also no significant difference in all metrics between the deep learning methods in the G60s and G150s groups (*p* > 0.05). In the G150s group, there was no significant difference between the conventional method and the deep learning methods in the LC and NC metrics (*p* > 0.05). Detailed data are shown in the Additional file 1: Table S3.

## Discussion

This proof-of-concept study demonstrates that the use of artificial intelligence techniques can effectively improve the quality of short-term scanning images. Images synthesized by the network model fusing CT prior information (p3DNet) had higher average image quality and lower regional SUV bias and variance than short-term scan PET images, images synthesized by the conventional processing method (NLM), and images processed by the model that does not consider CT prior information (3DNet) (Figs. 3, 4, 5, 6 and Additional file 1: Fig. S4). This finding suggests the value of introducing CT images with rich anatomical structure information into imaging models.

Considering that most of the equipment already configured in the nuclear medicine department of the hospital (such as desktop workstations) is not specifically enhanced for parallel computing, determining the ultimate visual effect of images often requires a relatively large network, which will result in having insufficient computing resources when running on these devices. Therefore, in this study, we designed a lightweight multimodal network that is easy to clinically test while achieving the image quality needed for diagnosis. In addition, our work mainly explores the use of CT information in PET/CT scans to improve the quality of PET images in short scanning situations.

The quantitative and semiquantitative results show that the quality of the synthesized images gradually increases with increasing scanning time, and the quantitative (SSIM, PSNR) and semiquantitative (SUVmax, SUV SD) values of the images synthesized by the model incorporating CT prior information are the closest to the full-time reference images for the same short-term scan duration. The PET images generated by 3DNet and p3DNet show similar trends in terms of correlation coefficients (Table 2). However, p3DNet outperforms 3DNet in different dose situations due to the introduction of CT prior information, which enhances the correlation in terms of structural information. The improvement of p3DNet in structural information is also validated in subjective evaluation, as shown in Fig. 8 and Fig. 9 for G6S to G15S.

From the subjective evaluation, the deep learning methods (3DNet and p3DNet) have a clear advantage in suppressing the noise of the G6s to G60s groups, while the model involving the fusion of CT prior information (p3DNet) is more advantageous in recovering the anatomical structure (Fig. 8, Fig. 9).

Previous studies have explored the utilization of CT prior information in low-dose PET image reconstruction [39], which used the anatomical boundary information from CT images as a regularization term for PET imaging to improve image quality. However, due to the limitations of the technology at that time, the extraction of CT edge information was manually designed and could not fully extract the deep information in CT. A recent study used MR as prior information in PET/MR multimodal imaging to improve PET image quality. The network structure is similar to the U-Net architecture, which directly concatenates MR and PET images as the input to the network without considering the fusion of high-dimensional features [27]. However, it has been indicated that feature fusion in higher dimensions is helpful in improving image quality [40]. Our network utilizes a lightweight 3D U-Net architecture (3D U-Net ensures consistency across slices) and performs simultaneous multimodal information fusion in both low and high dimensions (the number of parameters in our network is approximately 4 M, compared to the number of parameters in the standard 3D U-Net framework, which is approximately 30 M) to provide anatomically rich PET images. Our lightweight network is easier to deploy and test on PET/CT devices without significantly enhanced computational resources, and it has the potential for clinical practicality.

There are several limitations to our study. The ^18^F-FDG data used to train and test the deep learning model were obtained in a single hospital with a limited number of cases. Therefore, further studies with sufficiently large datasets from multiple medical centers are needed. Whether there are performance differences in network models trained on data from patients with different ages, weights, and reasons for scanning and how the data distribution information can be used to improve the performance of the models likewise need to be further investigated.

Data for a total of 270 pediatric patients of different ages and sexes were used in this retrospective study, and K-fold cross-validation was used to compensate for the lack of training samples to improve the generalizability of the network model. Although there was no model overfitting on the evaluation dataset, the lack of real clinical samples may result in overfitting for the application of the model in new cases, so a more accurate network model needs to be obtained after collecting more samples for training. Our study is based on a conventional ^18^F-FDG injection protocol that may not extrapolate to other tracers, such as ^18^F-NaF, ^18^F-FET, and ^68^Ga-PSMA. Due to limited data on small lesions, a lesion detection rate subgroup analysis was not performed for different lesion sizes. More studies are needed to investigate the effect of lesion shape, volume, and so on on the detection rate.

In medical applications that concern human health and life, AI technology must be used with caution because at present, AI networks have unclear operating mechanisms and are used as black boxes that cannot be explained by rigorous mathematical formulas. Fortunately, many research teams are studying the interpretability of AI, and over time, it is believed that AI networks will be rationally explained, improving safety in the use of AI models in medical clinics. Of course, most of the current applications of AI in the clinic are auxiliary and not directly involved in diagnosis, but AI can help doctors make predictions or preclassify their cases and reduce their workload. For the network proposed in this paper to be applied in clinical practice, 270 cases of patient data are not sufficient, and more patient data are needed to verify our findings.

## Conclusions

Based on the quantitative, semiquantitative and qualitative results, we can see that the enhancement of total-body PET/CT ultrashort-time scan images using artificial intelligence techniques and fusing prior CT information can significantly improve the image quality, which can help guide methods for shortening the patient's on-PET scan time, which is very promising for clinical diagnostic applications in easy-to-move pediatric patients. Despite the good performance of the proposed method, its safety needs to be extensively verified in clinical applications.

### Supplementary Information

**Additional file 1**. **Fig. S1**. Distribution of age, weight and sex of the 150 children included in the training dataset. **Fig. S2**. Distribution of age, weight and sex of the 120 children included in the evaluation dataset. **Fig. S3**. Semiquantitative metrics (Liver SD and Lesion SD) compared among different methods and scan times. **Fig. S4**. Bland‒Altman analysis of SUVmean differences compared among different methods and scan times. **Table S1**. Examples of injected activity for administration of 18F-FDG for torso imaging. **Table S2**. Average scores of the different subjective metrics. **Table S3**. Kruskal‒Wallis rank-sum test and Tukey’s post hoc test for multiple comparisons of different methods for different scan-time groups. **T1**: Detailed description of the structure of 3DNet.

## Data Availability

The datasets generated during and/or analyzed during the current study are available from the corresponding author upon reasonable request.
