# Supplementary material for: Reducing pediatric total-body PET/CT imaging scan time with multimodal artificial intelligence technology
Source: EJNMMI Phys. 2024 Jan 2;11:1. doi: 10.1186/s40658-023-00605-z (PMC10761657; doi:10.1186/s40658-023-00605-z)
Supplement: Supplementary file 1 — Additional file 1. Fig. S1. Distribution of age, weight and sex of the 150 children included in the training dataset. Fig. S2. Distribution of age, weight and sex of the 120 children included in the evaluation dataset. Fig. S3. Semiquantitative metrics (Liver SD and Lesion SD) compared among different methods and scan times. Fig. S4. Bland‒Altman analysis of SUVmean differences compared among different methods and scan times. Table S1. Examples of injected activity for administration of 18F-FDG for torso imaging. Table S2. Average scores of the different subjective metrics. Table S3. Kruskal‒Wallis rank-sum test and Tukey’s post hoc test for multiple comparisons of different methods for different scan-time groups. T1: Detailed description of the structure of 3DNet. [file 40658_2023_605_MOESM1_ESM.docx]

**Reducing pediatric total-body PET/CT imaging scan time with multimodal artificial intelligence technology**

**Running title:** Reducing pediatric total-body PET/CT imaging scan time with multimodal AI

**Authors:** Qiyang Zhang^1,#^, Yingying Hu^2,#^, Chao Zhou^2#^, Yumo Zhao^2^, Na Zhang^1^, Yun Zhou^3^, Yongfeng Yang^1^, Hairong Zheng^1^, Wei Fan^2^, Dong Liang^1^, Zhanli Hu^1,^*

**Affiliations:**

^1^ Lauterbur Research Center for Biomedical Imaging, Shenzhen Institute of Advanced Technology, Chinese Academy of Sciences, Shenzhen 518055, China.

^2^ Department of Nuclear Medicine, Sun Yat-sen University Cancer Center, Guangzhou 510060, China.

^3^ Central Research Institute, United Imaging Healthcare Group, Shanghai 201807, China.

**^#^First author:** Qiyang Zhang, Yingying Hu and Chao Zhou contributed equally to this work.

**^*^Corresponding author:** Zhanli Hu. Email: zl.hu@siat.ac.cn.

**Other’s emails:**

Qiyang Zhang: qy.zhang@siat.ac.cn Yingying Hu: huyy@sysucc.org.cn

Chao Zhou: zhouchao@sysucc.org.cn Yumo Zhao: zhaoym@sysucc.org.cn

Na Zhang: na.zhang@siat.ac.cn Yun Zhou: yun.zhou@united-imaging.com

Yongfeng Yang: yf.yang@siat.ac.cn Hairong Zheng: hr.zheng@siat.ac.cn

Wei Fan: fanwei@sysucc.org.cn Dong Liang: dong.liang@siat.ac.cn

**Supplementary Information**

**Figures**

Fig. S1. Distribution of age, weight and sex of the 150 children included in the training dataset. (a) Age and sex distribution. (b) Weight and sex distribution.


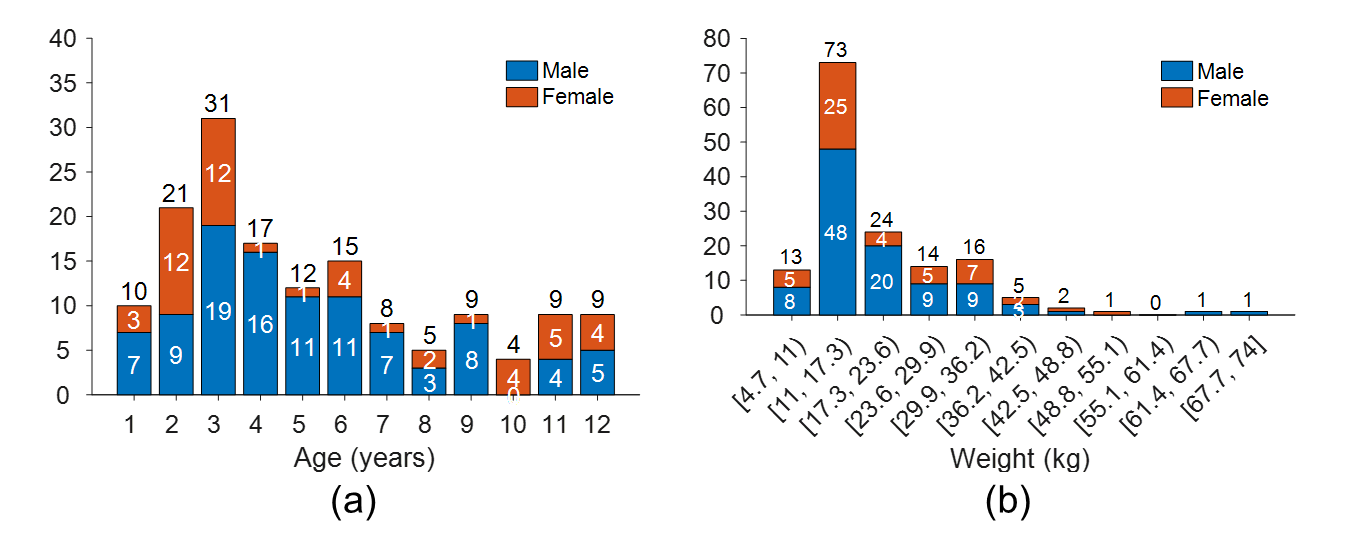


Fig. S2. Distribution of age, weight and sex of the 120 children included in the evaluation dataset. (a) Age and sex distribution. (b) Weight and sex distribution.


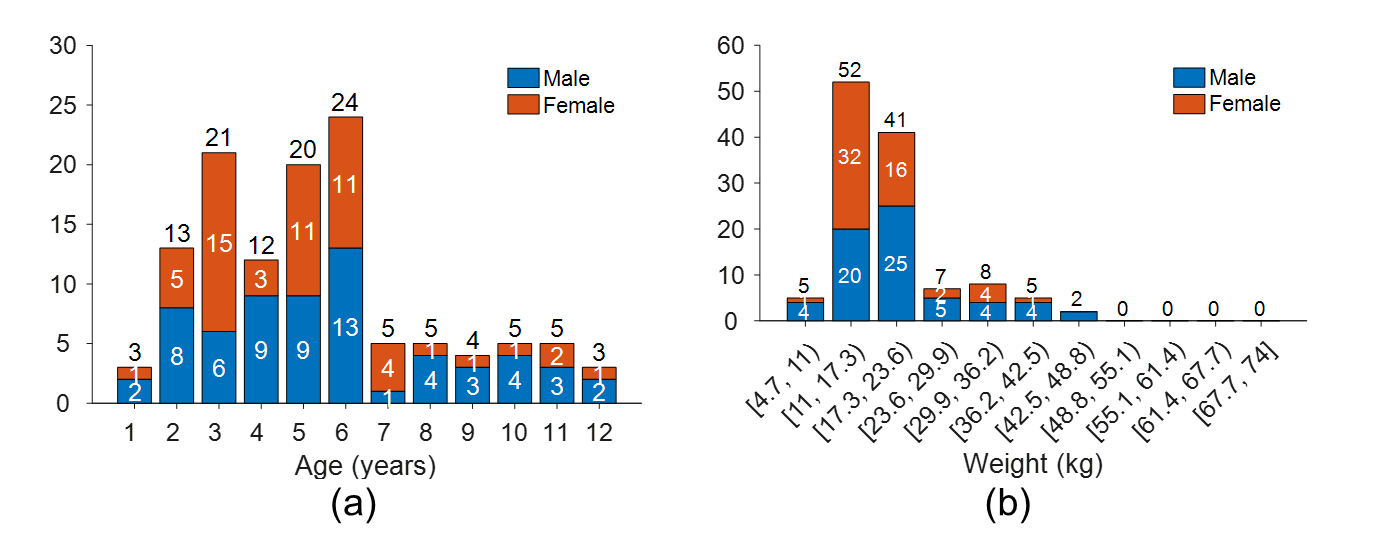


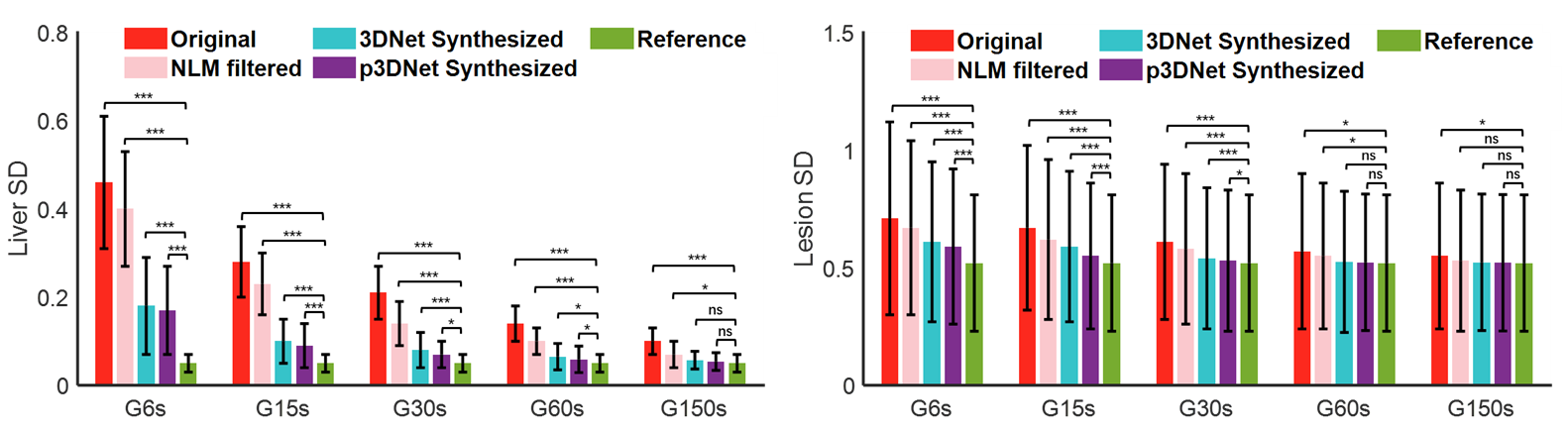


Fig. S3. Semiquantitative metrics (Liver SD and Lesion SD) compared among different methods and scan times. *, ***, and ns represent p < 0.05, p < 0.001, and nonsignificant, respectively.


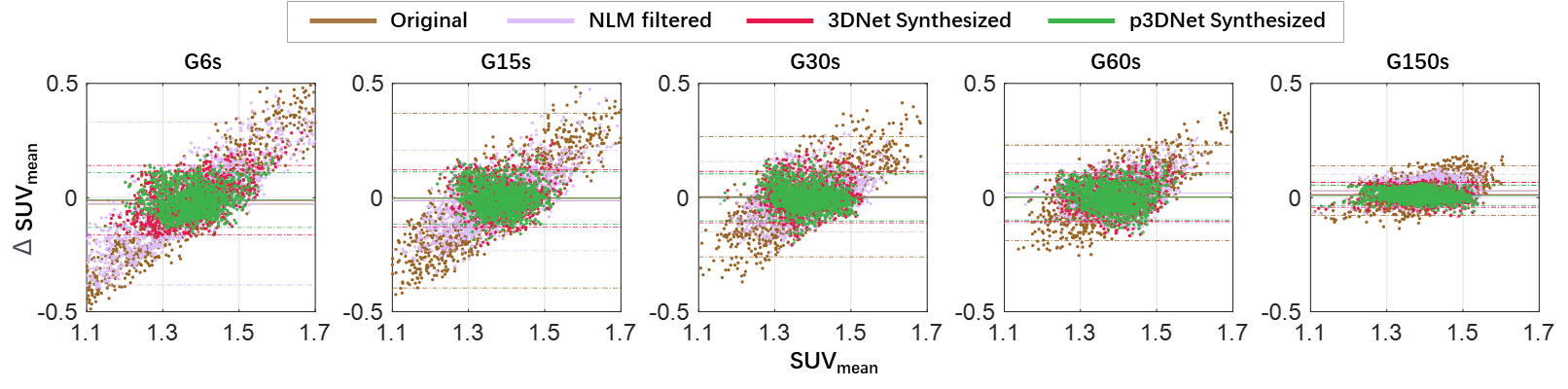


Fig. S4. Bland‒Altman analysis of SUVmean differences compared among different methods and scan times (all datasets).

**Tables**

**TABLE S1** Examples of injected activity for administration of ^18^F-FDG for torso imaging

|  | Example weight (kg) | Example Injected activity (MBq/kg) |
| --- | --- | --- |
| Neonate (1 m) | 5 | 3.89 |
| Child (1 y) | 10 | 3.34 |
| Child (3 y) | 14 | 3.51 |
| Child (5 y) | 17 | 3.81 |
| Child (7 y) | 20 | 3.88 |
| Child (9 y) | 25 | 3.77 |
| Child (11 y) | 35 | 3.70 |

|  | **G6s** | | | | **G15s** | | | | **G30s** | | | | **G60s** | | | | **G150s** | | | |
| --- | --- | --- | --- | --- | --- | --- | --- | --- | --- | --- | --- | --- | --- | --- | --- | --- | --- | --- | --- | --- |
|  | **Ori** | **NLM** | **3DNet** | **p3DNet** | **Ori** | **NLM** | **3DNet** | **p3DNet** | **Ori** | **NLM** | **3DNet** | **p3DNet** | **Ori** | **NLM** | **3DNet** | **p3DNet** | **Ori** | **NLM** | **3DNet** | **p3DNet** |
| **AC^*^** | 1.4 | 1.6 | 2.1 | 2.4 | 1.6 | 1.9 | 2.6 | 4.0 | 2.0 | 2.6 | 4.4 | 4.7 | 3.4 | 3.6 | 4.7 | 4.9 | 4.5 | 4.7 | 4.9 | 5.0 |
| **LC^*^** | 2.1 | 3.1 | 3.6 | 4.2 | 2.5 | 3.3 | 4.2 | 4.5 | 3.3 | 3.5 | 4.4 | 4.7 | 4.6 | 4.9 | 4.9 | 5.0 | 4.8 | 4.9 | 5.0 | 5.0 |
| **IN^*^** | 1.0 | 1.4 | 4.3 | 4.4 | 1.4 | 1.9 | 4.5 | 4.9 | 2.1 | 2.4 | 4.8 | 4.9 | 3.6 | 4.6 | 4.9 | 5.0 | 4.6 | 4.9 | 5.0 | 5.0 |

**TABLE S2** Average scores of the different subjective metrics (two readers)

*: **AC**: anatomical conspicuity, LC: lesion conspicuity, IN: image noise.

**TABLE S3** Kruskal‒Wallis rank-sum test and Tukey’s post hoc test for multiple comparisons of different methods for different scan-time groups.

| **G6s** | | | | **G15s** | | | **G30s** | | | **G60s** | | | **G150s** | | |
| --- | --- | --- | --- | --- | --- | --- | --- | --- | --- | --- | --- | --- | --- | --- | --- |
|  | **AC^*^** | **LC^*^** | **IN^*^** | **AC^*^** | **LC^*^** | **IN^*^** | **AC^*^** | **LC^*^** | **IN^*^** | **AC^*^** | **LC^*^** | **IN^*^** | **AC^*^** | **LC^*^** | **IN^*^** |
| **Ori vs. NLM** | 0.0039 | 0 | 0 | 0.0202 | 0 | 0.0137 | 0.0015 | 0.0077 | 0.0034 | 0.0125 | 0 | 0 | 0 | 0 | 0 |
| **Ori vs. 3DNet** | 0 | 0 | 0 | 0 | 0 | 0 | 0 | 0 | 0 | 0 | 0 | 0 | 0 | 0 | 0 |
| **Ori vs. p3DNet** | 0 | 0 | 0 | 0 | 0 | 0 | 0 | 0 | 0 | 0 | 0 | 0 | 0 | 0 | 0 |
| **NLM vs.3DNet** | 0 | 0 | 0 | 0 | 0 | 0 | 0 | 0 | 0 | 0 | 0.0150 | 0 | 0 | 0.8608 | 0.8390 |
| **NLM vs. p3DNet** | 0 | 0 | 0 | 0 | 0 | 0 | 0 | 0 | 0 | 0 | 0.0492 | 0 | 0 | 0.7593 | 0.0744 |
| **3DNet vs. p3DNet** | 0.0044 | 0 | 0.0672 | 0 | 0.0045 | 0.092 | 0.0247 | 0.0068 | 0.9352 | 0.0526 | 0.2063 | 0.8376 | 0.5138 | 0.9973 | 0.2826 |

*: **AC**: anatomical conspicuity, LC: lesion conspicuity, IN: image noise.

**Text**

**T1: Detailed description of the structure of 3DNet (comparison with p3DNet).**

The reference 3D neural network (3DNet) is shown in Fig. S5. The main frameworks of 3DNet and p3DNet are the same except for the prior information fusion block.

Fig. S5. Schematic diagram of the 3D convolutional neural network (3DNet). The main frameworks of 3DNet and p3DNet are the same except for the prior information fusion block. The arrows indicate the flow of computational operations, and the number of input and output feature images for the module is marked below the box.


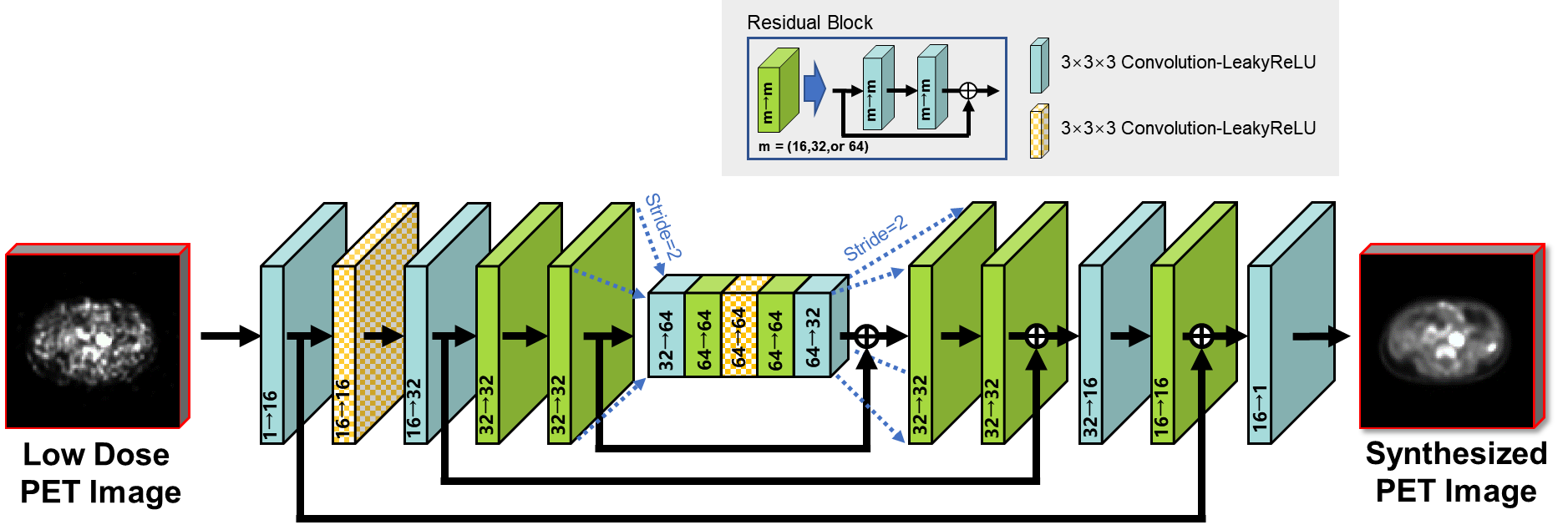


3DNet uses the same parameters as p3DNet, as follows. The input and output of the network were multislice data of size H × W × S, where H and W denote the image height and width, respectively, and S denotes the depth of successive adjacent multislice data, which was fixed to 5. The encoder and decoder part consisted of 3D convolutional layers (using 3×3×3 filters) and a leaky rectified linear unit (LeakyReLU) activation function. The number of channels is labeled below each box in Fig. S5. The downsampling operation was implemented by a convolutional layer with stride=2. Correspondingly, the upsampling operation was implemented by a deconvolutional layer that scaled the image size by a factor of two. Skip connections were applied between the residual module in the network and the encoder-decoder layer at symmetric positions to preserve the feature information.

The loss function, initial learning rate, training epochs, optimizer and training strategy of the 3DNet network are the same as those of p3DNet.
